# Central autonomic network-heart interplay in anorexia nervosa. A cross-spectral dynamic causal modeling study

**DOI:** 10.1016/j.nicl.2026.103980

**Published:** 2026-02-26

**Authors:** Monica Di Giuliano, Roberta Maria Lorenzi, Andy Schumann, Karl-Jürgen Bär, Feliberto de la Cruz

**Affiliations:** a Lab for Autonomic Neuroscience, Imaging and Cognition (LANIC), Department of Psychosomatic Medicine and Psychotherapy, Jena University Hospital, Jena, Germany; b Department of Brain and Behavioral Sciences, University of Pavia, Pavia, Italy

**Keywords:** Anorexia nervosa, Dynamic causal modeling, Central autonomic network, Neurovisceral integration model, Effective connectivity, Heart rate

## Abstract

The Central Autonomic Network (CAN) crucially maintains the homeostatic integrity of brain–heart communication, yet its directed interactions with cardiac control in anorexia nervosa (AN) remain poorly understood. To this end, we investigate the causal connectivity characterizing the CAN in 26 AN patients and 40 healthy controls, using a cross-spectral Dynamic Causal Modeling applied to resting state functional magnetic resonance imaging (fMRI). A Parametric Empirical Bayes framework was leveraged to estimate CAN connectivity group level differences and their linear association with group diagnosis and heart rate (HR). In patients, group connectivity differences revealed stronger top-down causal signaling from frontal/insular nodes to hypothalamus, alongside weaker connectivity from the anterior cingulate and insula to the hypothalamus and brainstem, respectively. These alterations may reflect bodily and emotional signals maladaptive processing at rest. In controls, HR was positively associated with most of the CAN connectivity, including with amygdala to prefrontal area causal influence: this might reflect a bottom-up role of amygdala in interoceptive signals processing, in absence of emotionally salient demands. In AN group, HR was negatively associated with most of the CAN connectivity, except for the connection from prefrontal to amygdala: in AN, a greater prefrontal control may emerge as a form of top-down compensatory regulation of limbic activity, at rest. Nevertheless, between-group CAN-HR differences were not found in the prefrontal-amygdala circuit. Instead, in patients, stronger top-down modulations encompassing frontal-insular and brainstem circuits resulted in driving maladaptive CAN-heart dynamics, compared to controls.

## Highlights

• CAN regulates brain–heart communication in anorexia nervosa (AN)
• Effective CAN-heart connectivity in AN is investigated with cross-spectral DCM.
• HR positively relates with bottom-up amygdala-prefrontal connection in controls.
• HR positively associates with top-down prefrontal-amygdala causal modulation in AN.
• Compared to controls, HR positively links with prefrontal-insular influence in AN.

## 1 Introduction

Emotional, cognitive and behavioral regulation mechanisms rely consistently on bidirectional brain-body communication, particularly through the Central Autonomic Network (CAN), which play a pivotal role in orchestrating the homeostatic and allostatic integrity of the body (Benarroch, 1993, Beissner et al., 2013, Ma et al., 2024, Quadt et al., 2022, Smith et al., 2017). This network is part of an internal regulation system controlling visceromotor, neuroendocrine, and behavioral responses, including different interconnected cortical and subcortical structures, i.e. the anterior cingulate, the insula, the ventromedial prefrontal cortices, the central nucleus of the amygdala, the hypothalamic nuclei and the brainstem regions (Benarroch, 1993, Beissner et al., 2013, Smith et al., 2017). These components are organized hierarchically and operate as a nonlinear dynamical system, supporting adaptive, goal-directed behavior (Quadt et al., 2022). The activity of the CAN is state dependent and thus sensitive to initial cognitive and emotional environment demands (Sklerov et al., 2019). Cortical structures (insular, cingulate and prefrontal areas) are involved in top-down high-level autonomic control mechanisms, while amygdala primarily coordinates emotional processing functions (Quadt et al., 2022). Brainstem and hypothalamus regulate physiological functions, including cardiovascular and gastrointestinal activity, respiration, and micturition (Benarroch, 1993, Cechetto and Shoemaker, 2009, Dampney et al., 2003, Fowler et al., 2008, Sklerov et al., 2019). The bidirectional interplay with the cardiovascular system is one of the most critical functions of the CAN, especially its regulation of heart rate (HR) through autonomic branches at the sinoatrial node (Thayer and Lane, 2009, Uijtdehaage and Thayer, 2000). This modulation is also reflected in heart rate variability (HRV), which is a vital index of parasympathetic control (Kim et al., 2018, Ma et al., 2024, Mayhugh et al., 2018, Smith et al., 2017).

The Neurovisceral Integration Model (NIM) integrates the main emotional, cognitive and autonomic functions exercised by the CAN as well as the interplay between the CAN and the cardiovascular system (Devinsky et al., 1995, Thayer and Lane, 2000, Thayer and Sternberg, 2006). According to this theoretical framework, the prefrontal, cingulate, and insula cortices form an interconnected network with bidirectional communication with the amygdala, which is under tonic inhibitory control via prefrontal-vagal signal transmission. Under normal circumstances, the prefrontal cortex identifies safety cues from the environment and exert its inhibitory control over sympathoexcitatory subcortical circuits, including the central nucleus of the amygdala (Ellis and Thayer, 2010, Thayer et al., 2009). Thus, hypoactivation of prefrontal areas disrupts this balance, leading to disinhibition of subcortical structures and chronic defensive states like hypervigilance and rumination (Park et al., 2013, Thayer et al., 2009). In this context, while low HR and higher resting HRV are linked to effective cognitive and flexible emotional responses, higher HR and lower resting HRV are associated with hypoactive prefrontal regulation. These evidences, in combination with hyperactive subcortical structures, would lead to maladaptive cognitive and emotional self-regulation mechanisms (Friedman, 2007, Hansen et al., 2003, Park et al., 2012, Ruiz-Padial et al., 2003, Thayer et al., 2009, Thayer et al., 2012).

Alterations in CAN function have been observed in various medical and neuropsychiatric conditions such as epilepsy, depression, anxiety, and schizophrenia (Kim and Whalen, 2009, Johnstone et al., 2007, Lewis et al., 2005, Sklerov et al., 2019, Thayer and Lane, 2009). Studies indicated that autonomic nervous system dysfunctions characterize transversally the spectrum of eating disorders (ED): baseline or rest observations, for example, evidenced predominantly a parasympathetic overactivation with correspondent sympathetic withdrawal, in ED patients (Buchhorn et al., 2016, Simões-Capela et al., 2019). Of these hallmarks, heart related indices are the most well-explored descriptors of autonomic functioning in ED, both at rest and in reaction to task stimuli (Mazurak et al., 2011, Simões-Capela et al., 2019). Nevertheless, limited research has been carried out on CAN dysfunctions in anorexia nervosa (AN), a severe mental disorder with the highest mortality rate among all mental health disorders (Arcelus et al., 2011, Papadopoulos et al., 2009). AN is characterized by restrictive eating behaviors, an overwhelming fear of weight gain, and significant body image distortions (Zhong et al., 2023). Markedly low body weight in relation to an individual’s developmental stage, as defined in the International Classification of Diseases (ICD-11), is one primary diagnostic criterion of the disease. Specifically, autonomic dysfunctions are present during the acute stage which persist during weight restoration (Jenkins et al., 2021, Lachish et al., 2009).

AN is generally associated with an higher HRV index and lower HR at rest, compared to controls (Bär et al., 2006, Melanson et al., 2004, Peyser et al., 2021, Spaulding-Barclay et al., 2016, Stein et al., 2012). Some studies in the field have proposed how these cardiovascular signatures might be potential consequences of prolonged starvation and caloric restriction at rest, leading to a predominant parasympathetic state to preserve energy in patients affected by AN (Peyser et al., 2021). Nevertheless, altered autonomic patterns have been reported to persist following partial or full weight restoration, indicating that aspects of autonomic imbalance may reflect disorder-related or trait-like characteristics rather than solely acute or chronic malnutrition (Lachish et al., 2009). In addition, disturbances in sympathetic–parasympathetic balance have been observed during refeeding and recovery phases, highlighting a complex and potentially maladaptive regulation of the autonomic nervous system that extends beyond starvation itself (Jenkins et al., 2021, Jenkins et al., 2022). Accordingly, converging neuroimaging findings further support this perspective, showing that even weight-restored individuals with AN exhibit persistent alteration in functional connectivity between regions implicated in central autonomic, interoceptive, and visceromotor processing, including nodes of the CAN and their interactions with large-scale cortical systems (e.g., de la Cruz et al., 2023b). Notably, while structural brain alterations have been proposed to normalize with weight gain, intrinsic connectivity abnormalities seem less sensitive to refeeding and may not represent purely BMI-dependent state markers, consistent with *meta*-analytic evidence reporting no robust association between resting-state brain activity and BMI in AN (Bernardoni et al., 2016, Bomba et al., 2015, Kaufmann et al., 2023, Mainz et al., 2012, Su et al., 2021). Together, these findings might suggest that although starvation plays a critical role in shaping autonomic function in AN, alterations in central autonomic network dynamics may contribute to sustained brain–heart dysregulation beyond the effects of malnutrition alone.

From this standpoint, the next step is to dive into the relationship between autonomic maladaptive mechanisms, as reflected in cardiovascular activity, and the neuronal changes that underlie these dysfunctions within the psychopathological profile of the disorder. In this direction, only few recent studies have dived into CAN-heart interplay disruptions in AN. De la Cruz et al. (2023a) reported overall reduced functional connectivity (FC) among core CAN regions in HR regulation, such as the ventromedial prefrontal cortex, anterior insula, amygdala, and anterior cingulate cortex, in acutely ill AN patients (Fassino et al., 2004, Khalsa et al., 2018, Lapidus et al., 2020, Pollatos et al., 2008). Further research in weight-restored AN patients showed adrenergic stimulation led to FC reductions between CAN and broader frontoparietal, autonomic and sensorimotor cortical areas (de la Cruz et al., 2023b). These FC disruptions notably correlated with higher anxiety, depression, and negative body image, indicating impaired interoception and visceromotor regulation (de la Cruz et al., 2023b).

A deep understanding of the causal hierarchical interactions between key units of the CAN-heart interplay at rest in AN is a timely scientific goal not yet investigated. To move beyond correlational FC analysis, the current investigation turns to effective connectivity, which quantifies the causal influence (i.e., the effect) between neuronal regions (Friston et al., 2003, Park et al., 2018, Penny et al., 2010). This involves estimating the model parameters that best explain observed Oxygen Level Dependent (BOLD) signal in resting state and task-designs approaches (Friston et al., 2003, Penny et al., 2010, Park et al., 2018). One of the methods mostly implemented to infer causal network interplays is Dynamic Causal Modeling (DCM). DCM relies on Bayesian inference methods by estimating the posterior values (i.e., the strength of the effective connectivity) based on observational data (e.g., blood oxygenation level-dependent-BOLD signal; Friston et al., 2003). So far, only Ma et al. (2024) have used DCM to link effective connectivity patterns in the CAN to HRV and HR in healthy individuals, at rest. Specifically, effective connectivity from the amygdala to the anterior cingulate and ventrolateral prefrontal cortex was negatively associated with HRV and positively with HR, highlighting that the bottom-up causal pathway from amygdala to higher autonomic hubs might underscore a potential circuit involved in cardiodynamics regulation, in resting conditions.

**Fig. 1:**
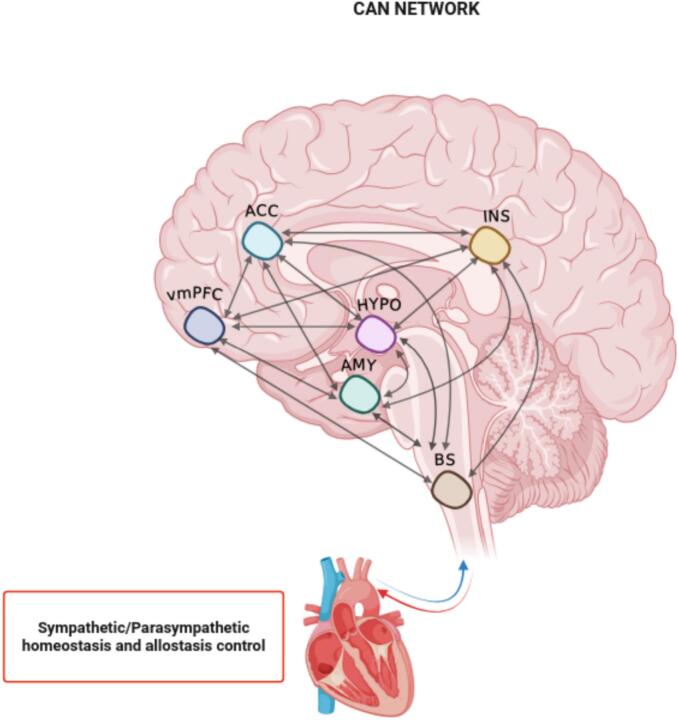
Neurovisceral Integration Model revised. Adaptation of the Central Autonomic Network (CAN) network conceptualization, based on the Neurovisceral Integration Model (NIM). The CAN is characterized by key cortical-subcortical regions reciprocally interconnected to each other, encompassing: ventromedial prefrontal cortex (vmPFC), anterior cingulate cortex (ACC), anterior insula (INS), amygdala (AMY), hypothalamus (HYPO) and brainstem (BS). This figure was created with BioRender (BioRender.com).

Building on the NIM framework (Fig. 1), the present study aims to model causal connectivity among CAN hubs in AN patients and controls. To this end, we exploratively focused on how heart rate − one representative metric of the overall CAN-cardiovascular balance − modulates prefrontal-subcortical interactions at rest. The aim of this study is to determine whether the hierarchical inhibitory processes described in NIM can be observed at rest in AN, providing a holistic characterization, together with previous findings, of the disorder effects on CAN network. (Thayer and Lane, 2009, Thayer et al., 2012). In order to test these hypotheses, we implemented a cross-spectral DCM analysis (sp-DCM). With respect to resting-state fMRI, sp-DCM has been found to be more accurate and more sensitive to group differences compared to time series stochastic DCM (Friston et al., 2016). This approach allows us to model neuronal effective connections that predict the observed statistical dependencies between regions, by estimating coupling strengths among brain regions (Friston et al., 2014).

This study aims to further characterize the hierarchical neuronal organization of the CAN by comparing directed CAN connectivity—and its linear association with resting HR—between patients and healthy controls, thus building a “bridge” between neuronal and autonomic state-related factors endorsing the perpetuation and maintenance of the disorder. This work constitutes one of the first attempts to map CAN–HR coupling using cross-spectral DCM, providing mechanistic insights that can inform future investigations regarding the association between autonomic and pathological profile of the disorder.

## 2 Materials and method

### 2.1 Participants

We recruited 31 women with AN and 44 age- and gender-matched HCs. All patients met DSM-IV criteria for AN, established via the Structured Clinical Interview for DSM-IV Axis I Disorders (SCID-I; First and Pincus, 2002), administered by board-certified psychiatrists in the treatment unit where patients were hospitalized. General psychopathology was further assessed using validated self-report questionnaires, including the Eating Disorder Inventory-2 (EDI-2; Thiel et al., 1997). Intellectual functioning was estimated with the Mehrfach-Wortschatz-Test B (MWT-B; Neumann and Wolfram, 1978). Screening for additional psychiatric conditions (e.g., major depressive disorder, obsessive–compulsive disorder, personality disorders) was conducted using SCID-I modules supplemented by a structured clinical interview performed by the same trained clinicians. In the HC group, the SCID-I confirmed the absence of any current or past psychiatric diagnosis. We have also taken arrhythmia, hypertension and other cardiac abnormalities into account among the exclusion criteria for the control group. Additionally, cardiac abnormalities, as measured by electrocardiogram (ECG), were routinely checked at the ward where patients were hospitalized. Regarding the AN group, the sample clinical characteristics can be examined more closely in Results section 3.1, where a comprehensive overview of the final sample analyzed for this study is presented.

All participants provided informed written consent, and the study was conducted with the approval of the local Ethics Committee of the University Hospital Jena and in accordance with the guidelines of the Helsinki Declaration (from 2013).

### 2.2 MRI data acquisition

MRI data were collected on a 3 T whole-body system equipped with a 64-element head matrix coil (MAGNETOM Prisma, Siemens Healthcare, Erlangen, Germany). During scanning, participants were instructed to keep their heads still and relax with their eyes closed without falling asleep or having systematic thought, in a dimly lit room. All scans were scheduled either in the morning or in the early afternoon. Importantly, the distribution of morning vs. afternoon sessions was balanced across AN and HC groups, preventing systematic circadian bias (Johnston et al., 2014). To minimize post-prandial autonomic fluctuations, participants were instructed to refrain from eating for at least two hours prior to scanning (Riganello et al., 2019). We acquired T2*-weighted images using a multiband multislice single-echo GE-EPI sequence with the following parameters: repetition time (TR) = 961 ms, echo time (TE) = 34.6 ms, flip angle (FA) = 90°, multiband factor = 6 with SENSE parallel imaging, matrix size = 98 x 98, field of view (FOV) = 196 x 196 mm^2^ with in-plane resolution 2 x 2 mm^2^ and 72 contiguous transversal slices of 2 mm thickness (interleaved), phase-encode direction posterior-to-anterior (j-), bandwidth per pixel = 18.2 kHz, and effective echo spacing = 0.56 ms. The session lasted approximately 12 min and contained a series of 750 whole-brain volume sets. The first four images were discarded to ensure a steady-state tissue magnetization condition. In addition to functional data, we also acquired high-resolution anatomical T1-weighted volume scans (MPRAGE) in sagittal orientation. MP-RAGE parameters were: TR = 2400 ms, TE = 2,34 ms, inversion time (TI) = 1,04 ms, FA = 8°, matrix size = 256 x 320, voxel size = 0,7 x 0,7 x 0,7 mm^3.^ The total number of slices was 320.

### 2.3 rs-fMRI preprocessing

Data analysis was performed using SPM12 (https://www.fil.ion.ucl.ac.uk/spm). Preprocessing steps of the fMRI data included slice timing correction, motion correction, alignment to the anatomical scan, and warping to the Montreal Neurological Institute template (MNI-12), with a resampling voxel size of 2 mm x 2 mm x 2 mm. Thus, normalized functional images in MNI space were smoothed with a 4-mm full-width half-maximum Gaussian kernel, to increase the signal to noise ratio. Quality control for excessive amounts of spatial displacement due to head motion was conducted by excluding subjects who had either (a) a mean FD greater than 0.30 mm, or (b) a frequency of occurrence of large FDs (i.e., FDs greater than 0.30 mm) > 20 percent of the total number of FDs in the run (Power et al., 2012). Only one control subject was excluded as not satisfying quality control criteria.

### 2.4 Region of interest definition and time series extraction

During the postprocessing, a general linear model (GLM) was applied to the time series of each individual dataset. The covariates of no interest included the six rigid body motion parameters, white matter, and cerebrospinal fluid signals. Low-frequency signal drifts were filtered using a 128-s high-pass filter. In the next step, the BOLD time series was extracted for six predefined regions of interest which could resemble the CAN system (Thayer and Sternberg, 2006). This procedure allowed us to extract subject-wise, the volumes of interest (VOIs) which represent the CAN regions, namely: the ventromedial prefrontal cortex (vmPFC), the anterior cingulate cortex (ACC), anterior insula (INS), amygdala (AMY), hypothalamus (HYPO), and nucleus of the solitary tract as representing brainstem (BS) (Fig. 1). Subject-specific peak were identified within group-level peak for each subject, following previous literature studies guidelines (de la Cruz et al., 2019, Mizzi et al., 2024, Razi et al., 2017, Rison et al., 2024, Zhou et al., 2016). Thus, each subject-specific peak was used as center of a geometrical sphere representing the VOIs underlying the CAN network; the regions with the corresponding MNI coordinates and radius of the spheres were: vmPFC = 0 44––14 (8 mm); ACC = 0 47 11 (8 mm); INS = −41 3 6 (8 mm); AMY = −24––4 −18 (5 mm); HYPO = −2––4 −10 (5 mm); BS = −3––43 −55 (3 mm).

Regarding the hemispheric lateralization, this study acknowledges that there is longstanding evidence suggesting right-hemispheric dominance for sympathetic and arousal-related autonomic regulation, especially considering task-based designs. Nevertheless, our focus in this study is specifically on parasympathetic-dominant resting-state dynamics, given current studies on the CAN autonomic system branches involvement due to lateralization asymmetries (Quadt et al., 2022). Because this study models resting-state cross-spectral effective connectivity rather than probe lateralized autonomic control or sympathetic responses to salient (e.g., emotional) stimuli, we opted for a constrained left-lateralized architecture to reduce model complexity and ensure stable DCM inversion. This also allowed us to examine autonomic regulatory features that are particularly relevant in the clinical profile under investigation, especially considering resting state parasympathetic autonomic dominance at rest, in AN (Peyser et al., 2021, Simões-Capela et al., 2019).

### 2.5 Heart rate measurement

We recorded cardiac signals at 500 Hz during a 10-minute resting period in the scanner using a 158 MR-compatible BIOPAC MP150 polygraph (BIOPAC Systems Inc., Goleta, CA, USA). A photoplethysmography (PPG) sensor was attached to the proximal phalanx of the left index finger. The PPG can be used as a surrogate technique for the ECG and is usually preferred to ECG systems for cardiac recordings since ECG-derived signals exhibit greater susceptibility to electromagnetic and biologic interference (Birkholz et al., 2004, Subasi, 2019). To reduce MRI-related and motion artifacts, we digitally filtered the PPG signal at 0.05–3 161 Hz. HR assessment was performed by detecting pulse waves offline to calculate the mean heart rate over the 10-minute resting period. Two control subjects were excluded from the final stage of DCM analysis as no HR data were available for these individuals. Ultimately, we chose to focus on HR as a proxy of cardiovascular activity as some evidence indicates that HRV measurements taken in an MR scanner during brain imaging instead of a standardized assessment in a laboratory could differ significantly, in comparison to HR indices (e.g. Schumann et al., 2021).

### 2.6 sp-DCM: First level analysis

The DCM analyses were conducted using DCM12 implemented in the Matlab-SPM12. The DCM analysis was performed on 26 AN patients and 40 healthy controls.

The first level analysis implies the state space model specification and estimation. For Bayesian Model specification, we implemented a sp-DCM, which is more specific for the analysis of rs-fMRI data (Friston et al., 2014). Sp-DCM is a deterministic approach: spectral models rely on second order statistics (i.e., cross spectra) of original time series, estimating their varying hidden states by estimating their time invariant covariance (Friston et al., 2014). The mechanism of sp-DCM could be described as follows (Ma et al., 2024, Novelli et al., 2024). The effective connectivity (ECs) are organized into an N × N matrix (i.e., the VOIs extracted as detailed in section 2.4), where N is the number of regions and N x N the effective connections which were estimated by sp-DCM model inversion. Here, we analyzed the effective connectivity of a network comprising 6 regions (i.e., nodes) resulting in 36 parameters to be estimated through model inversion. The effective connectivity quantifies the causal influences as the rate of change in neuronal activity per second (i.e., Hz; Zeidman et al., 2019a) and it is computed as

X(t)=Ax(t)+v(t)

where x(t) = [x1(t), …, x4(t)]T is a column vector of hidden neuronal states for the six regions, whose activity depends on between and on endogenous fluctuations, denoted by v(t). A is a 6x6 matrix with unknown effective connectivity parameters in units of Hz, to be estimated given the experimental recordings, namely the fMRI data (Novelli et al., 2024). A positive EC indicates that one node is increasing the activity in another one (excitatory effects); on the other hand, a negative (inhibitory effects) EC indicates that one node is decreasing the activity in a second node (Ma et al., 2024, Zeidman et al., 2019a). Diagonal connections (self-connections or endogenous fluctuations) in the A matrix are unitless, modeling the log scaling of parameters controlling self-inhibition within each node, determining their responsiveness to inputs from other nodes (Zeidman et al., 2019a). Self-connections are by design inhibitory, ensuring stability of the dynamic system model by regulating the activity of each region (Ma et al., 2024, Zeidman et al., 2019a). A higher positive self-connection EC implies greater inhibition in the node, reducing its responsiveness to inputs from other DCM nodes. A negative self-connection in EC, instead, implies lower inhibition in the node, leading to relatively increased responsiveness to inputs from other DCM nodes (Zeidman et al., 2019a). Overall, self-connections are referred to as intrinsic connectivity and those between-regions as extrinsic connectivity. During Bayesian model specification step, we explored all the possible inter-intra modulations between each network by opting for a fully connected model (matrix A connection set from-to each node), which is based on NIM conceptualizations as shown in Fig. 1 (Thayer and Sternberg, 2006). This step was ultimately performed to allow a comparison between groups in terms of connectivity parameters differences. Then, we proceeded with the model inversion for sp-DCM to find the parameters posterior densities that offer the best trade-off between explaining the data and minimizing complexity (Friston et al., 2016). Ultimately, at the first‐level DCM analysis, we evaluated the quality of the modeling: this step has been performed by calculating the fraction of variance of empirical BOLD signals which can be explained by the variance of the simulated BOLD signals. That is, the best fit between empirical and simulated BOLD signals generated by the model. In line with literature, we applied a 10% threshold of the explained variance as a criterion for our subjects to qualify for DCM analysis (Zeidman et al., 2019b). As result, the percentage of explained variance of the model fell above 80%, leading to including all the subjects in the following second level analysis steps.

### 2.7 Parametric empirical Bayes: Second level analysis

After estimating a fully connected network with sp-DCM in AN patients and healthy control, we implemented a group-level DCM analysis using the parametric empirical Bayes approach (PEB) which consists of a hierarchical statistical model over connectivity parameters to quantify the commonalities and differences across subjects (Friston et al., 2016). The parameters of interest are collated and modelled at the second level using a GLM, with between-subject unexplained variability captured by a covariance component (Zeidman et al., 2019a). Thus, individual differences in connection strengths are decomposed into hypothesized group-level effects, plus any unexplained random effects (Zeidman et al., 2019a). Overall, PEB allows to achieve a multivariate normal probability density over each parameter, resulting in expected probability values for each parameter in the model: namely, a Bayesian posterior inference to test whether any effective connectivity were different from zero (Friston et al., 2003). The PEB approach differs fundamentally from other group-level connectivity modeling, since it is a hierarchical model introducing random effects on parameters. Specifically, it assumes that all subjects have the same basic architecture, but they differ in terms of the strength of connections within that model (Zeidman et al., 2019a). Furthermore, in PEB analysis the hypothesized between-subject effects are continuous behavioral or clinical measures rather than the presence or absence of connections (Zeidman et al., 2019a). Bayesian posterior probability (Bayesian-PP) was used to evaluate the reliability of each effective connectivity pattern between groups (Baig, 2020). Higher Bayesian-PP values indicate greater reliability (Zeidman et al., 2019a, Baig, 2020). Thus, we used sp-DCM to infer effective connectivity for each subject and then the PEB model was used to integrate subject-level results with group analysis. From the multivariate normal probability density computed over the parameters, two relevant information regarding the cross-spectral causal connectivity are extracted: the posterior expected values (Ep), which outline the average strength of connectivity across each group, and the and the posterior covariance (Cp) of each parameter, which quantify the level uncertainty (or inter-subject variability) in the parameter estimates.

The PEB analysis was performed in two main step analyses:

1. CAN baseline dynamics within and between groups. The within-groups analysis involved exploring how the CAN network causal dynamics are organized in patients and controls: This was achieved by specifying separately for both groups a design matrix which would include the group commonalities (average causal connectivity) across all the subjects. Then, we performed a separate PEB between-groups analysis to compute how the CAN system is characterized differently between patients and controls: To this end, we specified a design matrix in which the group differences − first regressor of interest − were encoded as group membership/diagnosis (1 for patients and −1 for controls)
2. Can-heart rate linear relationship within and between groups. The second step involved a within-group analysis to outline how heart rate is associated with each effective connectivity estimate, separately per each group: accordingly, the design matrix was built up by adding HR measurements as first regressor of interest. At the end, a separate PEB between-groups analysis was performed according to a final regressor of interest, which takes into account the main interaction effect between heart rate measurements and group membership/diagnosis. This latter analysis was ultimately performed to estimate the group differences in the CAN effective connections and heart rate linear relationship

All the regressors of interest following the first constant term were mean-centered, to model the mean changes in connectivity over subjects, and then add to or subtract from this the between-subject effects (Zeidman et al., 2019a). In each GLM, all DCM parameters were allowed to contribute to all group effects. We considered a Bayesian-PP as reliable if exceeding 0.99, corresponding to a Bayes factor of 4 indicating the highest statistical significance (Baig, 2020).

## 3 Results

### 3.1 Sample characteristics

Out of 75 initial participants who met the inclusion criteria, 9 were excluded as: 1 control subject did not meet the quality control criteria during the rs-fMRI preprocessing analysis step; for 1 control subject, HR data were not available; 5 anorexia patients met the criteria for atypical subtype (BMI > 18.5), as well as 1 control subject met the criteria for obesity BMI type (BMI > 30).

The final sample of subjects analyzed is composed by 26 AN patients and 40 healthy individuals. Due to variability in documentation and participants' refusal to provide information, as well as incomplete demographic and clinical data for some participants, the following available data concerning comorbidities (SCID-I) and medication were collected in the AN sample. Specifically: eleven AN patients had a depressive clinical profile in their medical history; two patients had social phobia disorder; three borderline personality disorder; one attention deficit hyperactivity disorder; finally, three post-traumatic stress disorder in comorbidity with anorexia; five patients were taking antidepressant medication; four antipsychotic medication; three both antidepressant and antipsychotic medication; finally, one patient was taking a stimulant for ADHD. The mean duration of illness is 8.3 years from the date of scanning. Finally, out of a total number of 66 subjects analyzed, 7 subjects were found to be left-handed (patients: n = 4; controls: n = 3).

Further, we found that anorexia patients and healthy controls were not statistically different accounting for age (AN = 24.07 ± 6.5; HCs = 22.9 ± 2.8, p = 1), but statistically different accounting for education (p = <0.001). Nevertheless, the two groups were not statistically different for general intelligence (IQ estimate) scores at the MWT-B (AN = 27.7 ± 4.5; HC = 28.6 ± 6.2, p = 0.60). The two samples were statistically different for BMI (AN = 15.8 ± 1.8; HCs = 22.5 ± 2.4, p = <0.001) and for clinical scores obtained at the EDI-2 questionnaire (AN = 320.3 ± 1.8; HCs = 234.8 ± 56.05, p = <0.001). Regarding heart measures, we found a statistically significant difference in HR (bpm) measurement between anorexia and healthy samples (AN = 65.06 ± 10,07; HCs = 74,1 ± 10,1, p = <0.001). Table 1 contains a summary of statistics, including mean values and standard deviations of anamnestic, clinical and physiological data.

**Table 1:** Demographics and clinical data. Mean ± standard deviation values and range are reported for demographic and clinical parameters.

	AN patients (n = 26)	Controls (n = 40)	Statistic^†^	p value
General				
Age (years)	24.07 ± 6.5 (18 to 50)	22.9 ± 2.8 (18 to 33)	U = 520	1
BMI (kg/m)	15.8 ± 1.8 (11.8 to 18.5)	22.5 ± 2.4 (18.6 to 28.3)	t = -12.12	< 0.001
Clinical and cognitive measures				
EDI-2 total score	320.3 ± 46.7 (216 to 414)	234.8 ± 56.05 (120 to 314)	U = 900	< 0.001
MWT-B	27.7 ± 4.5 (14 to 33)^a^	28.6 ± 6.2 (7 to 37)^b^	t = -0.51	1.00
Illness duration at the time of scanning				
< 2 years	n = 3			
from 2 to 5 years	n = 8			
from 5 to 10 years	n = 4			
> 10 years	n = 3			
N/A	n = 8			
Physiological data				
Heart rate (beats per minute/bpm)	65.06 ± 10.07 (35.5 to 82.2)	74.1 ± 10.1 (56.1 to 96)	t = -3.46	< 0.001
Education				
No	n = 0	n = 0	χ2 = 14.71	< 0.001
Primary	n = 0	n = 0		
Secondary	n = 9	n = 1		
Higher level	n = 16	n = 39		
N/A	n = 1	n = 0		
Physical activity (hours per week)				
from 1 to 2 h	n = 4	n = 9		
from 3 to 4 h	n = 4	n = 17		
from 5 to 6 h	n = 4	n = 1		
more > 6h	n = 1	n = 3		
N/A	n = 13	n = 10		
Last menstrual cycle period				
Same year of scanning	n = 11	n = 26		
1 year before scanning	n = 6	n = 1		
2 years before scanning	n = 0	n = 1		
3 years before scanning	n = 3	n = 0		
>3 years before scanning	n = 2	n = 0		
N/A	n = 4	n = 12		

^†^The distribution of all variables was checked using the Levene test, and group comparisons were performed using either *t*-test, Mann-Whitney *U* test, or Chi-square test (χ^2^) as appropriate. Illness duration refers to the time between reported illness onset and the MRI scan date.

^a^ Available in AN = 20.

^b^ Available in HCs = 28.

BMI = body mass index, HR = heart rate, EDI-2 = Eating Disorder Inventory-2, MWT-B = Mehrfachwahl-Wortschatz-Intelligenztest, N/A = not accessible data.

### 3.2 Results from sp-DCM

#### 3.2.1 CAN effective connectivity within and between groups

**Fig. 2:**
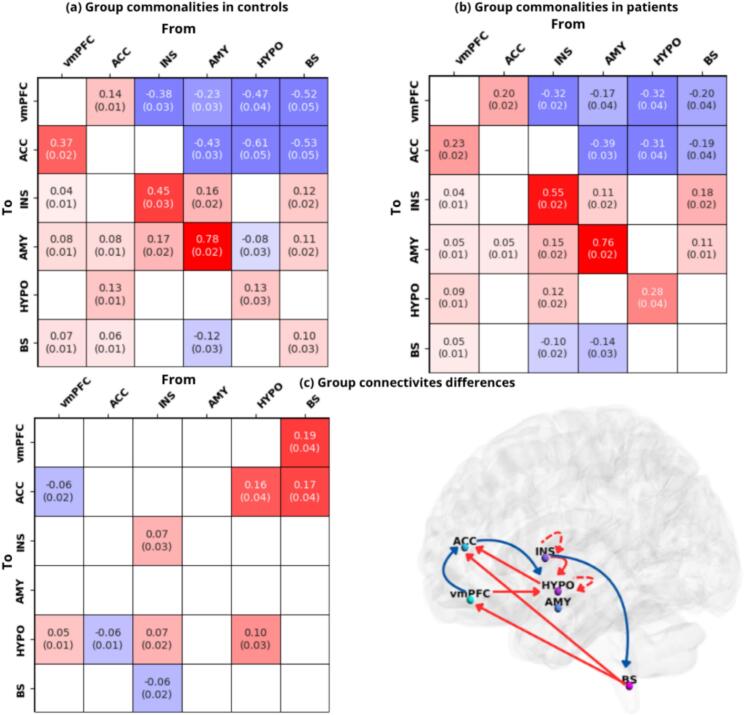
CAN baseline causal dynamics within and between groups (a) Intrinsic and extrinsic connectivity patterns in healthy controls and (b) intrinsic and extrinsic connectivity patterns in AN patients, in terms of group mean estimates. Here, positive values express excitatory connections while negative inhibitory connections. (c) Group differences in the CAN intrinsic and extrinsic connectivity, based on group membership. Here the color gradient denotes positive values for those connections which are stronger in patients than controls (positive linear relationship with the covariate), and negative values for those couplings which are less strong in patients than controls (negative linear relationship with the covariate). The group estimate differences are mapped onto a brain template. The solid arrows indicate directed connectivity from one node to the other, while the dashed arrows denote self-connections. When interpreting each matrix, self-connections should be accounted as having a different scale unit, as log-scaling unitless of the estimated parameters, constrained to be inhibitory by the model. Only the connections with a posteriori probability of more than 99% (PP) are displayed. In cases where the Bayesian-PP is less than or equal to 0, the connectivity element is displayed in white. The posterior expected values and the posterior level of covariance expressed in standard deviation (in parentheses) are shown. vmPFC = ventromedial prefrontal cortex; ACC = anterior cingulate cortex; INS = anterior insula; AMY = amygdala; HYPO = hypothalamus; BS = brainstem (NTS). All the regions are left lateralized.

Fig. 2 shows the effective connectivity intrinsic and extrinsic patterns accounting for the group mean of the DCM connections (group commonalities) separately for healthy controls and anorexia patients. At the same time, Fig. 2 displays the group effective connectivity differences based on group membership. Each of the parameters is depicted as a matrix element. To ease comprehension of the results in terms of how CAN is differently characterized in line with group membership, the group estimates differences are also mapped onto a brain template with BrainNet Viewer software (https://www.nitrc.org/projects/bnv/). The average connectivity strength and covariance for each effective connectivity parameter are displayed in each connectivity matrix, when the Bayesian-PP exceeds 0.99 (Zhou et al., 2018, Uscătescu et al., 2021).

Regarding the average connectivity patterns observed in healthy control group (Fig. 2a), we can observe that vmPFC and ACC receive mostly inhibitory causal influences from all the other nodes: exceptions are the connectivity patterns from vmPFC to ACC (0.37 Ep), as well as from ACC to vmPFC (0.14 Ep), which are reciprocally excitatory. Insula is observed to receive mostly excitatory causal modulations, except from ACC and HYPO, which resulted to be not significant. At the same time, AMY is the only node within the CAN which receives causal modulations from all the high-order and subcortical hubs: these connectivity patterns are shown as excitatory, except for the connection from HYPO (−0.08 Ep). Ultimately, hypothalamus is observed to receive only one excitatory influence from ACC (0.13 Ep), while all the other modulations result as not significant; brainstem, instead, is target of only two excitatory causal modulations from vmPFC and ACC, respectively (0.07 Ep, 0.06 Ep), except for the influence from AMY which is shown as inhibitory (−0.12 Ep).

Accounting for the average connectivity computed independently for anorexia nervosa patients (Fig. 2b), similarly to controls, we observed overall bottom-up inhibitory causal connectivity targeting mostly the vmPFC and ACC nodes, together with top-down/bottom-up excitatory causal influences on limbic (insula, amygdala) and hypothalamic/brainstem regions. Additionally, only in anorexia, we also outlined: an excitatory causal influence from vmPFC and INS to HYPO, respectively (0.9 Ep, 0.12 Ep); an inhibitory connection from INS to BS (−0.10 Ep); no significant connections from ACC to HYPO and BS, and from HYPO to AMY.

To reveal group differences in the CAN causal dynamics, we considered the linear relationship between group membership/diagnosis and CAN connectivity (Fig. 2c). The positive linear relationship between group diagnosis and CAN connections showed increased connectivity strength in patients compared to controls, from: vmPFC and INS to HYPO (0.05 Ep, 0.07 Ep, respectively); HYPO to ACC (0.16 Ep); BS to vmPFC and to ACC (0.19 Ep, 0.17 Ep, respectively). On the other hand, the negative linear relationship between group diagnosis and CAN connections showed decreased connectivity strength in patients compared to controls, from: vmPFC to ACC, ACC to HYPO and INS to BS (−0.06 Ep). No significant group differences were found for connections involving AMY, either as a source or target node. In the Supplementary materials, we reported the group average parameters computed across both groups, as resulting from the between-groups analysis (Fig. S1).

#### 3.2.2 Can-heart interplay within and between groups

**Fig. 3:**
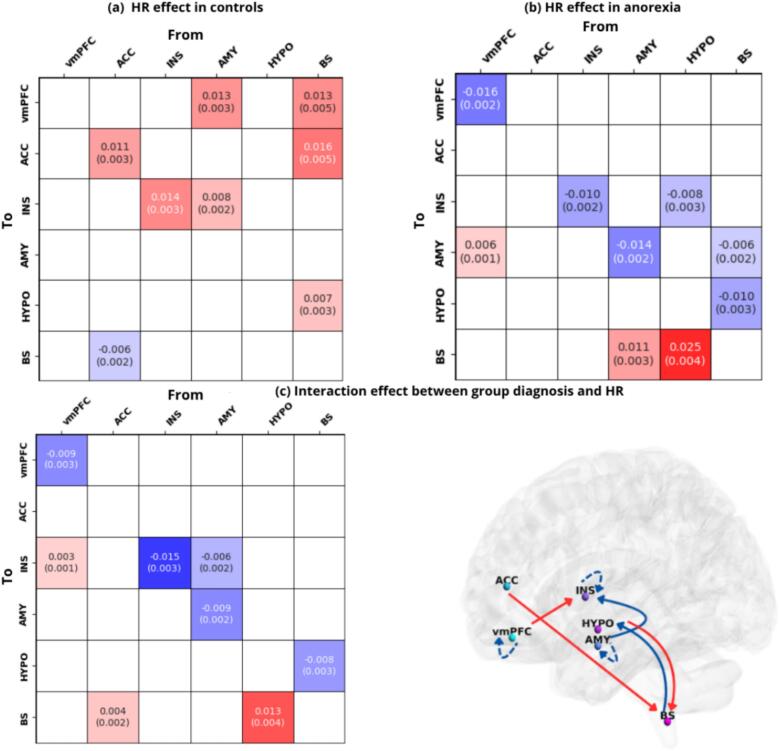
CAN-heart linear association within and between groups (a) Heart rate linear association with each connectivity parameter in controls. (b) Heart rate linear association with each connectivity parameter in patients. Here, positive and negative values expressed the positive or negative linear association of HR with each within-group intrinsic/extrinsic estimate. (c) Interaction effect between group membership and heart rate. Here the color gradient denotes positive values for those connections which are stronger in patients than controls in association to HR (positive association), and negative values (negative association) for those couplings which are less strong in patients than controls in association to HR. The group estimate differences are mapped onto a brain template. The solid arrows indicate directed connectivity from one node to the other, while the dashed arrows denote self-connections. When interpreting each matrix, self-connections should be accounted as having a different scale unit, as log-scaling unitless of the estimated parameters, constrained to be inhibitory by the model. Only the connections with a posteriori probability of more than 99% (PP) are displayed. In cases where the Bayesian-PP is less than or equal to 0, the connectivity element is displayed in white. The posterior expected values for each DCM parameter are shown, together with the posterior level of covariance expressed in standard deviation (in parentheses). vmPFC = ventromedial prefrontal cortex; ACC = anterior cingulate cortex; INS = anterior insula; AMY = amygdala; HYPO = hypothalamus; BS = brainstem (NTS). All the regions are left lateralized.

Fig. 3 displays the within-groups HR linear association with each intrinsic/extrinsic effective connection in patients and controls together with the group differences in the connectivity strength patterns, resulting from the main interaction effect between group membership/diagnosis and HR measures. The average connectivity strength values, together with the covariance in terms of standard deviations, are shown for all the analyses, for PP > 0.99 (Zhou et al., 2018, Uscătescu et al., 2021).

In controls (Fig. 3a), we observed an overall prominence of positive linear relationship between HR and most CAN causal connectivity. Specifically, a positive association between HR and the connectivity from AMY and from BS to vmPFC can be observed (0.01 Ep): higher HR has been shown to be associated with greater causal connectivity stemming from amygdala-brainstem nodes to prefrontal one. A reverse pattern of linear relationship between HR and the connection between ACC and BS can be observed: while HR is positively associated with the connectivity strength from BS to ACC (Ep = 0.01), it is negatively associated with the effective connectivity pattern from ACC to brainstem (Ep = -0.006). Thus, lower HR is demonstrated to be associated with greater causal modulation stemming from cingulate node to brainstem. At the same time, a positive association with HR can be shown for the effective connectivity strength from AMY to INS (0.008 Ep), as well as from BS to HYPO (0.007 Ep).

In anorexia patients (Fig. 3b) we showed an overall predominance of negative linear relationship between HR and most of the CAN causal connectivity. Specifically, lower HR has been demonstrated to associate with greater causal modulations from: HYPO to INS (−0.008 Ep), BS to AMY (−0.006 Ep) and to HYPO (−0.01 Ep). Instead, we observed few positive associations between HR and the connectivity strength, notably from vmPFC to AMY (0.006 Ep), as well as from AMY and from HYPO to BS structure (0.01 Ep, 0.02 Ep, respectively).

The group commonalities results arising from the within groups PEB analysis − on the effect of HR computed separately in controls and patients − are detailed in the Supplementary Materials (Fig. S2a, S2b).

Finally, as regards the main interaction between group membership/diagnosis and HR with each CAN connectivity (Fig. 3c), we showed that − in comparison to controls − patients are characterized by an increased connectivity strength (positive linear relationship) in the following pathways: from vmPFC to INS (0.003 Ep); from ACC and HYPO to BS (0.004 Ep, 0.01 Ep, respectively). Moreover, we observed decreased connectivity strength (negative linear relationship) from AMY to INS (−0.006 Ep) and from BS to HYPO (−0.008 Ep), in patients compared to controls.

The group commonalities computed across both samples, resulting from the interaction effect group x HR analysis (Fig. S2c), are detailed in Supplementary materials.

For sake of completeness, a set of supplementary analyses was conducted to improve transparency and assess the potential influence of clinical and physiological factors on the observed brain–heart findings. Specifically, we explored whether effective connectivity within the central autonomic network (CAN) differed as a function of psychotropic medication use, psychiatric comorbidities, and bradycardia (Fig. S3a–c). In addition, we tested whether patient subgroups defined by medication use or comorbidities were comparable in terms of BMI and illness duration using independent-samples *t*-tests (Fig. S4). We further performed exploratory brain–behavior association analyses by correlating individual-level posterior estimates from subject-specific DCMs with clinical variables (BMI, symptom severity, and age), using Pearson correlations with false discovery rate (FDR) correction, following established approaches in the field. These analyses did not reveal statistically systematic and significant effects of these factors on CAN effective connectivity. All supplementary analyses are reported in full in the Supplementary materials.

## 4 Discussion

In the present study, we performed DCM analyses on resting state fMRI data to investigate the relationship between CAN causal connectivity and heart rate, in AN patients and healthy controls.

When considering the CAN baseline dynamics in both groups independently, we found that both forebrain and hindbrain structures included into CAN (amygdala, insula, hypothalamus, brainstem) exert overall inhibitory bottom-up influences on vmPFC and ACC. On the other hand, other forebrain structures including insula, amygdala and hypothalamus, receive mostly top-down and bottom-up excitatory causal modulations, while brainstem was primarily a target of top-down excitatory causal influences. These patterns might support an adaptive CAN functional hierarchical organization at rest, which can be described in three main layers (Sklerov et al., 2019). First, brainstem governs reflexive autonomic control and integrates stress, pain, and sleep responses; second, at the forebrain level, hypothalamus integrates these overall neuroendocrine and autonomic functions. Lastly, forebrain structures serve as bottom-up and top-down relays of autonomic mechanisms: together with vmPFC, anterior limbic circuit nodes (ACC, amygdala and insular cortices) coordinate bodily signals with goal-related autonomic responses (Aleksandrov et al., 2022, Benarroch, 1993, Sklerov et al., 2019). Overall, reciprocal ascending and descending projections between brainstem and forebrain structures are essential to map autonomic processes depending on the environmental needs, sustaining a homeostatic autonomic state (Aleksandrov et al., 2022, Valenza et al., 2025). Based on these findings, a different autonomic connectivity pattern emerges in resting state conditions compared to task studies testing the NIM (Thayer et al., 2009). First, ascending inhibitory projections from brainstem could regulate forebrain structures (vmPFC, ACC), preventing unnecessary goal-directed cognitive/emotional responses in absence of external salient stimuli. In parallel, anterior limbic circuit nodes (amygdala, insula) and hypothalamus might contribute to modulate vmPFC and ACC, establishing an overall autonomic visceromotor and emotional balance at rest (Benarroch, 1993, Sklerov et al., 2019, Valenza et al., 2025). In this context, the absence of emotionally salient stimuli might have favored bottom-up processing of bodily signals stemming from brainstem and limbic circuit layers to frontal/cingulate nodes, at rest (Etkin et al., 2011, Ma et al., 2024). Ultimately, the excitatory descending projections from forebrain structures (vmPFC, ACC) within the limbic circuit and to brainstem could allow mapping brainstem and hypothalamic activity, to co-orchestrating and balancing the regulation of bodily signals processing (Benarroch, 1993, Isaacson and Scanziani, 2011, Shadlen and Newsome, 1994, Sklerov et al., 2019).

Between-group analysis on the CAN causal organization revealed significant differences in key CAN connectivity strengths. In comparison to controls, patients show stronger top-down modulations stemming from vmPFC and insula to hypothalamus. These connectivity patterns likely relate to disorder symptoms, as these pathways are central to stress sensitivity and cortical regulation of food intake (Clarke et al., 2023, Kaye et al., 2011, Roger et al., 2022, Wu et al., 2020). In addition, we showed less strong top-down modulations from cingulate/insular nodes to hypothalamic/brainstem ones, respectively. Neuroimaging studies have linked cingulate-hypothalamic connectivity patterns to disrupted reward and appetite signals integration in eating disorders (Ahima and Antwi, 2008, Alfano et al., 2020, Etkin et al., 2011, Frank et al., 2016, Stratford and Kelley, 1999). Moreover, given the role of ACC and insula in the salience network and interoception processing, these causal pathways might reinforce a broader maladaptive processing of body dependent interoceptive and physiological states signals in patients, coming from brainstem/hypothalamus (Boehm et al., 2014, Kerr et al., 2016, Lamotte et al., 2021, McFadden et al., 2014). AN patients also demonstrated a significantly weaker causal connectivity from vmPFC to ACC. According to some studies, the vmPFC can modulate hypothalamic activity either directly or via the ACC, particularly under stress (Clarke et al., 2023, Frank et al., 2016). This overall circuit might further reinforce distorted interoceptive awareness and food intake avoidance behaviors in patients (Clarke et al., 2023). Moreover, the functional connectivity between vmPFC and ACC emerging from literature supports the balance between cognitive control and self-referential processes (Esposito et al., 2018, Quadt et al., 2022). In AN, however, these connections may contribute to internally focused and rigid self-control, including voluntary suppression of food intake despite physiological hunger signals (Cowdrey et al., 2011, Jacobs et al., 2014, Seidel et al., 2016). Overall, we propose that CAN group differences spotlight how top-down causal influences − exerted by higher autonomic hubs (e.g., vmPFC, ACC, INS) − might be related to impaired integration of bodily and emotional cues arising from hypothalamic and brainstem regions. These connectivity patterns might ultimately reinforce autonomic and emotional detachment from salient bodily states, building the fundamentals for an interoceptive and autonomic maladaptive resting state activity in patients (Lucherini et al., 2022).

When interpreting the present effective connectivity group differences findings, it is important to acknowledge that additional clinical factors may have contributed to the observed differences in group connectivity. In particular, the clinical history of the anorexia nervosa sample is relevant, given that depression was reported as the most common psychiatric comorbidity alongside eating-disorder symptoms in many patients. Previous studies have suggested that depressive symptoms have an additive effect in anorexia nervosa, whereby depression may modulate autonomic functioning. This includes relative parasympathetic dominance, as reflected in cardiovascular measures (Jelinek et al., 2019). Consistent with this framework, the present results revealed group differences in directed causal dynamics between higher-order CAN nodes and the hypothalamic region depending on group diagnosis (i.e. differences in connectivity strengths between the vmPFC/ACC/insular and the hypothalamic region). Previous functional connectivity studies in depression have demonstrated disrupted interactions within the limbic–hypothalamic axis, supporting the notion that altered prefrontal–limbic regulation in conditions characterized by impaired control inhibition (a trait defining AN) may influence autonomic functioning indirectly via hypothalamic and hypothalamic–pituitary–adrenal axis mechanisms. Ultimately, this could result in maladaptive cardiovascular signatures in patients characterized by depressive episodes (Agorastos et al., 2019, Herman et al., 2005, Zhou et al., 2020).

AN patients demonstrated to be characterized by significantly lower resting HR than healthy controls, in line with evidence that starvation and prolonged caloric restriction produce bradycardia as an energy-conserving adaptation (Bär et al., 2006, Melanson et al., 2004, Peyser et al., 2021, Spaulding-Barclay et al., 2016). Regarding CAN-heart causal interplay, we overall observed a positive association between HR and most the CAN connectivity in healthy individuals: specifically, higher HR is shown to be associated with greater bottom-up projections targeting forebrain regions (e.g., BS to vmPFC/ACC, amygdala to vmPFC and INS). Among these connections, of note is the frontal-amygdala causal pathway: we showed that higher HR is associated with greater bottom-up processing stemming from amygdala to frontal node, in controls. Amygdala is a key region in threat detection, fear conditioning, and emotional salience, and its reciprocal functional and structural feedback loops underlined the modulatory role of prefrontal area on limbic activity (Banks et al., 2007, Beauregard et al., 2001). It is worth noticing that the recruitment of frontal area modulatory activity becomes salient when individuals are actively engaged in emotional self-regulation processes. For example, a bidirectionality of the mPFC-amygdala causal connectivity has been demonstrated in healthy individuals during task conditions studies, leading controls to engage both top-down and bottom-up signaling to process fearful/emotionally salient stimuli (Rangaprakash et al., 2018). However, another study investigating the CAN-heart interplay in healthy individuals has highlighted a positive association between amygdala to prefrontal causal connectivity with HR, at rest (Ma et al., 2024). In this context, as no emotionally salient or threatening stimuli were presented, it is possible that prefrontal top-down regulation becomes less significant, in favor of a predominance of bottom-up processing stemming from amygdala. Accordingly, we might propose that resting condition might have strengthened the importance of amygdala − together with overall greater bottom-up projections − in orchestrating interoceptive information flow within the brain–heart axis (Craig, 2008, Critchley and Garfinkel, 2017, Etkin et al., 2011, Ma et al., 2024, Šimić et al., 2021).

Among the few negative associations between HR and CAN connectivity in healthy individuals, we outlined that lower HR is associated with greater top-down modulation stemming from cingulate to brainstem. As part of the anterior limbic system, ACC send descending projections to hypothalamic and brainstem nuclei, in order to sustain high order autonomic control of bodily states: this ultimately might serve regulating interoceptive predictions about the physiological and emotional state of the body (Critchley et al., 2003, Lamotte et al., 2021, Quadt et al., 2022). Specifically, ACC is implicated in cardiovascular modulatory changes, including contextual generation of arousal states necessary to meet cognitive, emotional and motor behavioral demands (Bush et al., 2000, Carter et al., 1999, Critchley et al., 2000, Critchley et al., 2003, Phan et al., 2002). Accordingly, the top-down autonomic signaling from ACC to brainstem may indicate the need of a well-coordinated control of cardiovascular arousal/self-regulatory activity at rest.

In AN patients, we observed that HR was significantly negatively associated with most of the key CAN hubs connectivity: notably, lower HR has been shown to be associated to greater bottom-up modulations targeting forebrain structures (e.g., HYPO to INS; BS to AMY; BS to HYPO). Furthermore, we demonstrated only few positive associations between HR and key CAN connections in patients, mostly encompassing top-down projections to brainstem (e.g., AMY to BS, HYPO to BS). The predominance of greater bottom-up causal influences on higher autonomic nodes and of greater top-down modulations targeting brainstem, might suggest a less predominant role of central CAN hubs in the autonomic CAN-heart axis control. This might be consistent with FC studies demonstrating that central forebrain CAN nodes might be less engaged in the cardiovascular dynamics control in AN patients, due to an overall reduced pattern of FC between these regions (de la Cruz et al., 2023a).

Interestingly, we noticed in patients a positive association between vmPFC to amygdala connection and HR (higher HR linked to stronger prefrontal-amygdala coupling). At first glance, this might not be in line with NIM studies: accordingly, the top-down tonic inhibition exercised by the prefrontal cortex over amygdala, serves to support flexible and appropriate responses to changing environmental demands, reflected by lower HR (Thayer et al., 2009). Ultimately, this top-down control is essential in order to promote effective emotional regulation and cognitive self-control (Chepenik et al., 2010, Fowler et al., 2017, Gee et al., 2013, Greicius, 2008, Ochsner et al., 2012, Phelps, 2006, Silvers et al., 2015, Valenza et al., 2025). By contrast, diminished prefrontal regulation and amygdala hyperactivation is associated with elevated HR, which correspond to maladaptive emotional and physiological states (Hansen et al., 2003, Park and Thayer, 2014, Ruiz-Padial et al., 2003, Thayer et al., 2009, Valenza et al., 2025). The interplay between prefrontal and limbic areas is particularly relevant when considered in the context of AN psychopathology, creating the fundamentals for maladaptive emotional regulation (Steward et al., 2022). Structural and functional alterations of amygdala in AN confirmed the role of this region in supporting an overall distorted negative emotional processing, along with other key psychopathology hallmarks encompassing food-related reward processing, body image, and rumination (Brockmeyer et al., 2019, Marcolini et al., 2024).

Several factors might be considered in order to interpret our findings. First, most of the studies on the NIM are task-based and FC oriented (Ma et al., 2024). Accordingly, we might propose that resting state might show different compensatory top-down prefrontal recruitment from task-driven regulation studies: this ultimately might not directly translate to adaptive autonomic outcome. In other words, it might be possible that in AN a greater top-down prefrontal engagement in regulating limbic activity emerges as an attempt of controlling amygdala heightened response, in association to autonomic arousal. This gains importance when considering that no active emotional self-regulation activity − leading to the emergence of prefrontal modulatory activity over limbic nodes − is required at rest (Banks et al., 2007, Beauregard et al., 2001). Ultimately, the causal connectivity pattern observed in patients might not lead to a successful tonic prefrontal modulation of limbic activity, reinforcing rigid cognitive-emotional control processes as well as maladaptive emotional regulation strategies which are characteristic of the psychopathology (Brockmeyer et al., 2019, Marcolini et al., 2024, Pauligk et al., 2025, Steward et al., 2022, Sudo et al., 2024). For example, most studies — predominantly task-based — have demonstrated that patients exhibit impaired and significant connectivity stemming from the prefrontal area to amygdala when exposed to salient stimuli (e.g. food or body stimuli). This results from excessive top-down cognitive control and the regulatory role of the prefrontal area over limbic system activity (e.g. Marcolini et al., 2024, Pauligk et al., 2025). At the end, future studies should test and replicate whether the higher top-down prefrontal involvement in regulating amygdala activity at rest might reflect an overall compensatory or maladaptive mechanism associated with autonomic and emotional functioning in AN.

When accounting for the interaction between group diagnosis and HR measures, no differences have been observed in the prefrontal-amygdala connectivity. However, we outlined greater top-down modulations from forebrain structures, − encompassing frontal, cingulate and hypothalamic areas, − targeting brainstem and insula regions, respectively, in anorexia compared to controls. The top-down modulation from vmPFC to insula might be linked to specific cardiovascular dynamics at rest in patients, compared to controls: anterior insula has been significantly associated with the regulation and modulation of heart rate, integrating cardiac signals with emotional and cognitive information, in concert with cingulate and prefrontal area (Craig, 2009, Critchley et al., 2002, Critchley et al., 2004, Jones et al., 2015, Menon and Uddin, 2010, Nogueira et al., 2025, Shaffer and Ginsberg, 2017). The connectivity between medial prefrontal cortex − key node of the default mode network − and anterior insula − key salience network node − has been shown to be involved in the balance of internal emotional states with external situational demands: these domains are particularly affected in AN (Seeley et al., 2007, Nogueira et al., 2025). In this context, our findings might strengthen how many of the symptoms of AN (e.g., distorted body image, hunger signals processing, disturbed interoceptive awareness) can be related to altered insula activity as well as default-insular connectivity (Boehm et al., 2014, Kappou et al., 2021, Pollatos et al., 2008).

## 5 Limitations and future directions

Caveats should be considered when interpreting our findings. First, we defined regions of interest in the left hemisphere only. The choice was motivated by the overall parasympathetic dominance characterizing autonomic functioning of AN at rest, as well as by the nature of the study, which did not involve probe lateralized autonomic control or sympathetic responses to salient stimuli (Peyser et al., 2021, Quadt et al., 2022, Simões-Capela et al., 2019). In this regard, there is longstanding evidence suggesting right-hemispheric and left-hemispheric dominance for sympathetic and arousal-related autonomic regulation and parasympathetic regulation, respectively. Accordingly, autonomic asymmetry has been reported with lateralized cortical and neurovascular/autonomic activity (Quadt et al., 2022). Nevertheless, these findings are inconsistent across studies and exploring inter-hemispheric differences in CAN connectivity was also beyond the scope of the present work. Future studies should address this aspect by incorporating bilateral cortical-subcortical CAN nodes in order to test how inter-hemispheric differences in the CAN affect connectivity patterns.

Second, the small sample size of our study could be accounted as a limitation. In this context, the sample size affects the comparison of the covariance or level of uncertainty values between the group commonalities values across both groups and the effect of each regressor of interest on some of the parameters. A higher number of participants would be beneficial to either lower the inter-subject variability range or either show a significantly different credibility interval between commonalities and regressor of interest. At the same time, a larger sample size would allow us to address another significant limitation of the current study. In this regard, we acknowledge that medication, clinical comorbidities and illness duration could account for the differences in neuronal causal coupling found in the present study. Accordingly, over the years, the focus of psychiatric research has shifted to investigating common biological markers across traditional diagnostic boundaries and stages of psychiatric illness severity, while considering different clinical mediating factors (Hickie et al., 2013, McGorry et al., 2007). For instance, studies have demonstrated the potential adverse effects of psychotropic medications on autonomic heart signatures. In the current study, the majority of medicated patients were treated with antidepressants, predominantly selective serotonin reuptake inhibitors, and/or second-generation antipsychotics, which are known to affect cardiovascular activity (Alvares et al., 2016, Sarlon et al., 2016).

Overall, in the context of ED, BMI, comorbidities, clinical history, illness duration and phase of the disease, as well as medications should be more closely addressed (Simões-Capela et al., 2019). Among these factors, age also plays an important role in affecting autonomic markers, including cardiovascular activity metrics (i.e., Abhishekh et al., 2013). Specifically, in the current study we focused on adulthood, as the samples presented a restricted age range: thus, we did not take into account how different neurodevelopmental stages of the disease might impact on the CAN-heart causal dynamics. In addition, even though less explored in the context of ED, handedness should be accounted for as another potential moderating factor when addressing CAN causal connectivity patterns. Handedness dominance might influence cardiovascular autonomic activity, as demonstrated by task studies investigating the association between autonomic heart-related responses and brain dominance (i.e., Barawade et al., 2023). Overall, we suggest that future studies with larger and more clinically stratified samples will be necessary to explicitly model the effects of these overall clinical and anamnestic factors, by disentangling their contributions to alterations in effective connectivity and brain–heart coupling in anorexia nervosa.

Thirdly, our VOIs were defined in advance based on previous studies of the CAN under the NIM theory (e.g. Thayer and Lane, 2000). This ROI-based approach enabled us to test specific, theory-driven hypotheses. However, it would be important to replicate these findings using complementary, data-driven approaches to further characterize differences in CAN-heart connectivity causality between patients and controls. This could potentially involve a larger number of nodes representing the CAN architecture, as well as considering different cortical-subcortical hubs and fine anatomical parcellations (e.g. anterior, mid and posterior insula subregions). Another limitation of the VOI time series extraction is the susceptibility of small autonomic structures, such as the NTS and hypothalamus, to physiological noise. High-frequency signals (e.g. cardiovascular and respiratory) can generate spectral components that overlap with or alias into the low-frequency range of resting-state fMRI, which could affect estimates of neuronal connectivity. Consequently, residual cardiac-related pulsatility cannot be fully excluded. Therefore, findings involving brainstem nuclei and the hypothalamus should be interpreted with caution, and future studies would benefit from including dedicated physiological noise correction methods (e.g. RETROICOR).

Fourth, it is important to point out that potential group differences in neurovascular coupling, particularly given the metabolic state of the AN group, should be accounted for. DCM has the advantage − in comparison to other functional connectivity or other effective connectivity methods − to jointly account and estimate both neuronal and hemodynamic parameters during the generative model of BOLD response inversion. Variational Bayesian estimation procedures can be exploited to estimate and compare separately neuronal parameters, neurovascular coupling parameters, and finally the parameters governing hemodynamics (e.g., Jafarian et al., 2019). Nonetheless, given the explorative nature of the present study, we did not conduct a separate and direct statistical comparison of hemodynamic parameters (e.g., transit time, decay) between groups. Future studies with a specific focus on hemodynamic variability—such as hierarchical modeling of HRF parameters— could be valuable to further clarify vascular versus neuronal contributions in this population.

In conclusion, we focused on HR as a representative biomarker of CAN–heart interplay, under the NIM framework. However, given the significance of HRV as a representative metric of brain–heart interplay in ED, notably in anorexia, future studies should consider other autonomic indices, such as HRV, to gain a better and comprehensive understanding of cardiovascular–autonomic causal dynamics (Jenkins et al., 2021, Simões-Capela et al., 2019).

## 6 Conclusions

Our causal connectivity approach revealed distinct patterns of CAN–heart interactions in healthy individuals versus AN patients. In comparison to healthy individuals, patients showed stronger causal influences encompassing vmPFC and INS to hypothalamus, as well as weaker ACC and INS to hypothalamus and to brainstem pathways, respectively. These causal influence patterns stemming from higher autonomic hubs and directed on hypothalamic/brainstem regions might underline disrupted interoceptive and visceromotor signals processing at rest, in patients. At the same time, accounting for HR association with each CAN connection separately per group, we observed that higher HR is associated with greater amygdala to prefrontal bottom-up processing in healthy individuals. This might ultimately serve an adaptive interoceptive and bodily signals processing at rest, in absence of emotionally salient stimuli. At the same time, in AN group we observed a positive association between HR and prefrontal to amygdala connection: this finding might be linked to the possibility that resting state favored the emergence of a top-down prefrontal effortful control on limbic heightened activity − as autonomic arousal increases − in terms of a potential compensatory or maladaptive cognitive-emotional mechanism. Finally, between-groups differences in the CAN-HR interplay revealed no significant differences in the prefrontal-amygdala pathway: instead, patients showed stronger top-down modulations stemming from vmPFC to insula, and from major autonomic hubs (ACC, hypothalamus) to brainstem, which might drive CAN-heart maladaptive dynamics in AN. Overall, these findings provide mechanistic insights into maladaptive interoceptive and autonomic regulation in AN, offering a potential neurobiological framework as a starting point to guide future clinical investigations targeting brain–heart dynamics.

## Ethical statement

Ethical approval for the involvement of human subjects in this study was granted by the local Ethics Committee of the University Hospital Jena and in accordance with the guidelines of the Helsinki Declaration (from 2013).

## CRediT authorship contribution statement

**Monica Di Giuliano:** Writing – review & editing, Writing – original draft, Visualization, Validation, Supervision, Software, Methodology, Investigation, Formal analysis, Data curation, Conceptualization. **Roberta Maria Lorenzi:** Writing – review & editing, Validation, Supervision, Methodology. **Andy Schumann:** Writing – review & editing, Validation, Supervision, Project administration, Methodology, Funding acquisition. **Karl-Jürgen Bär:** Validation, Supervision. **Feliberto de la Cruz:** Writing – review & editing, Validation, Supervision, Methodology.

## Funding

This work was partially supported by the DFG grant CR 994/2-1.

## Declaration of competing interest

The authors declare that they have no known competing financial interests or personal relationships that could have appeared to influence the work reported in this paper.

## Data Availability

Data will be made available on request.
