# Supplementary material for: Central autonomic network-heart interplay in anorexia nervosa. A cross-spectral dynamic causal modeling study
Source: Neuroimage Clin. 2026 Feb 26;49:103980. doi: 10.1016/j.nicl.2026.103980 (PMC12964038; doi:10.1016/j.nicl.2026.103980)
Supplement: Supplementary Data 1 [file mmc1.docx]

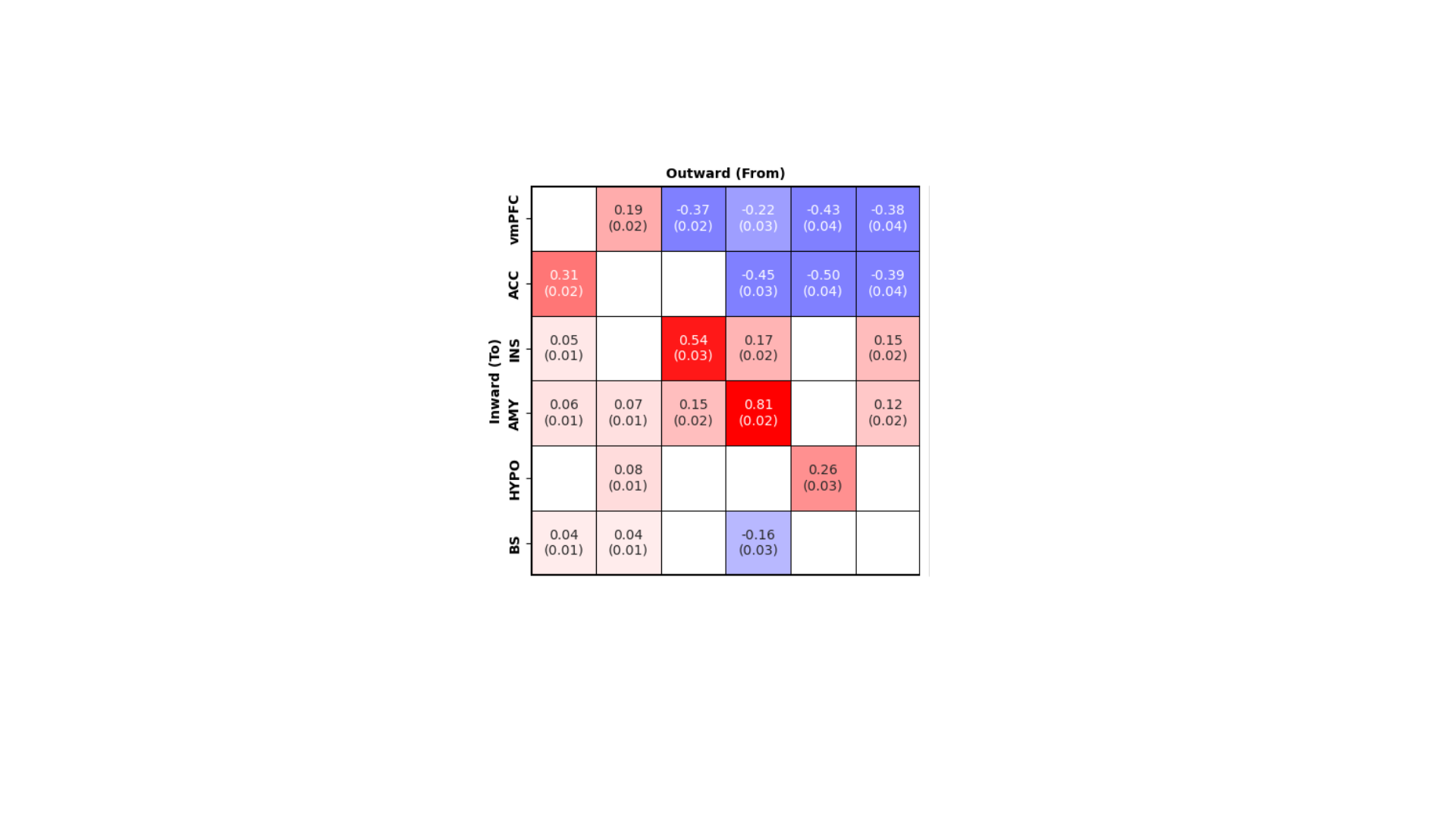


**Fig. S1 CAN group commonalities across both groups**. Group average connectivity resulting from the between-groups analysis on the effect of group membership on mean estimates. Positive values express excitatory while negative inhibitory connections, in average, across both groups. When interpreting each matrix, self-connections should be accounted as having a different scale unit, as log-scaling unitless of the estimated parameters, constrained to be inhibitory by the model. Only the connections with a posteriori probability of more than 99% (PP) are displayed. In cases where the Bayesian-PP is less than or equal to 0, the connectivity element is displayed in white. The posterior expected values (average connectivity) and the posterior level of covariance expressed in standard deviation (in parentheses) are shown in each connectivity matrix. Nodes: vmPFC = ventromedial prefrontal cortex; ACC = anterior cingulate cortex; INS = anterior insula; AMY = amygdala; HYPO = hypothalamus; BS = brainstem (NTS).


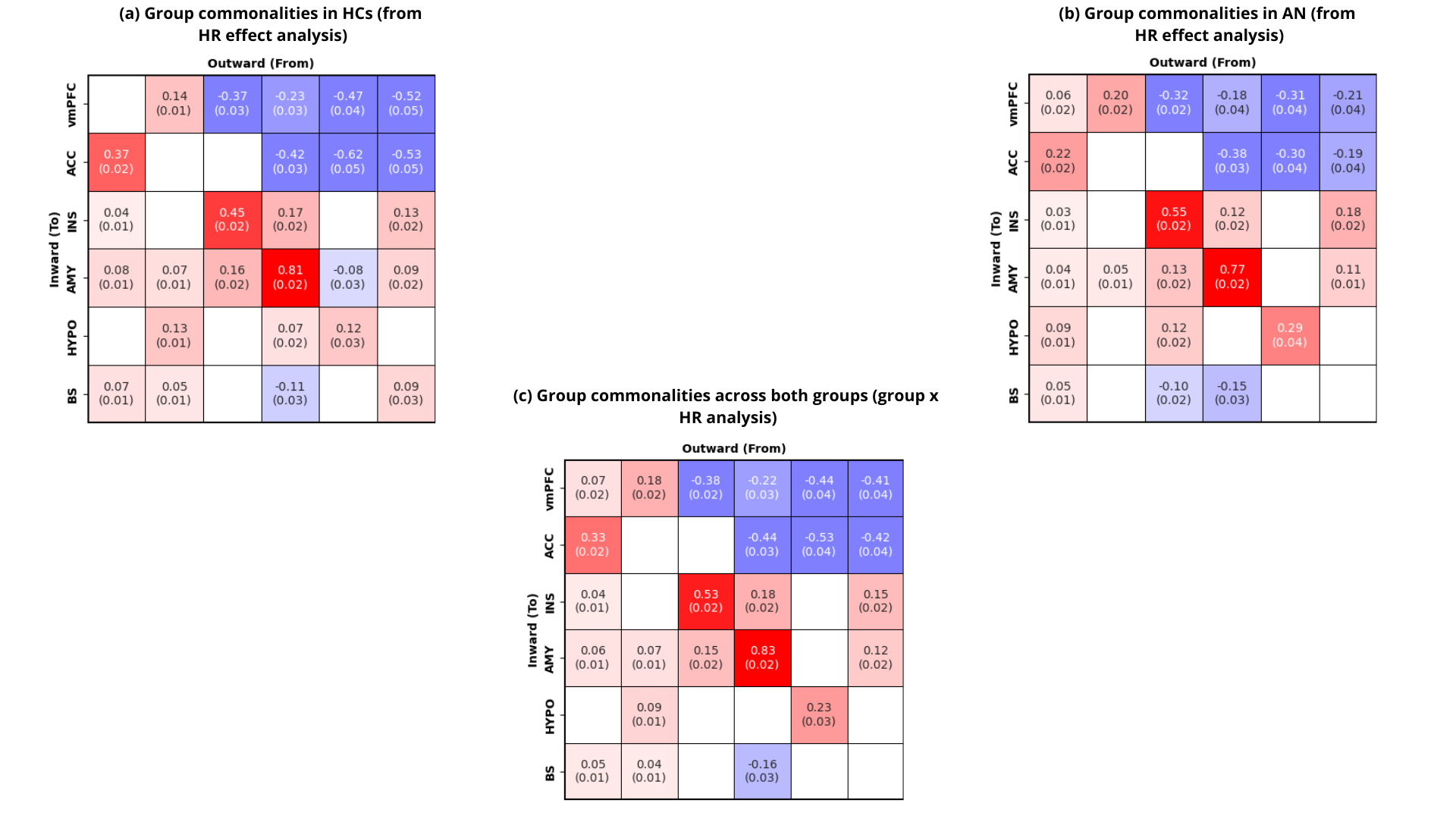


**Fig. S2 CAN-heart average connectivity within and between groups (a)** Group commonalities in HCs from the HR effect on within-subject PEB analysis. **(b)** Group commonalities in AN from the HR effect on within-subject PEB analysis. **(c)** Group commonalities across both samples from the interaction effect between group and HR on between-groups PEB analysis. Positive values express excitatory while negative inhibitory connections, in average, across both groups. When interpreting each matrix, self-connections should be accounted as having a different scale unit, as log-scaling unitless of the estimated parameters, constrained to be inhibitory by the model. Only the connections with a posteriori probability of more than 99% (PP) are displayed. In cases where the Bayesian-PP is less than or equal to 0, the connectivity element is displayed in white. The posterior expected values (average connectivity strength) and the posterior level of covariance expressed in standard deviation (in parentheses) are shown in each connectivity matrix. Nodes: vmPFC = ventromedial prefrontal cortex; ACC = anterior cingulate cortex; INS = anterior insula; AMY = amygdala; HYPO = hypothalamus; BS = brainstem (NTS).


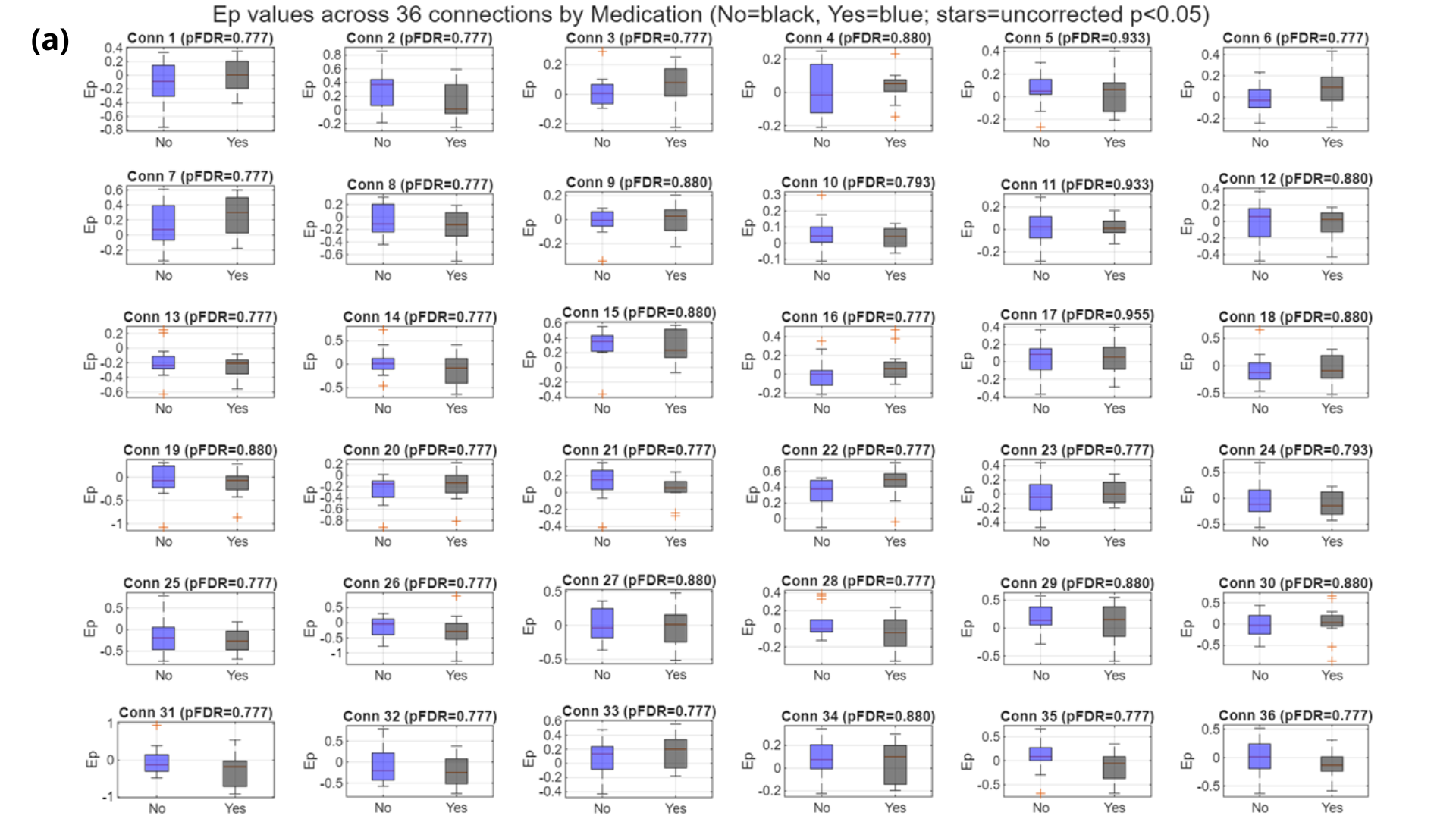


**Fig. S3a CAN connectivity changes in association to medication use.** Distribution of subject-level effective connectivity estimates (Ep) across all 36 CAN connections for patients treated with (n = 15, either alone or in combination with non-psychotropic drugs) and without (n = 11) psychotropic medication, in blue and black respectively. Group differences were assessed using independent two-sample t-tests for each connection, with false discovery rate (FDR) correction applied across the 36 tested connections for medication status. No connections survived FDR correction (pFDR ≥ 0.77). Boxplots illustrate the distribution of connectivity estimates across subgroups.


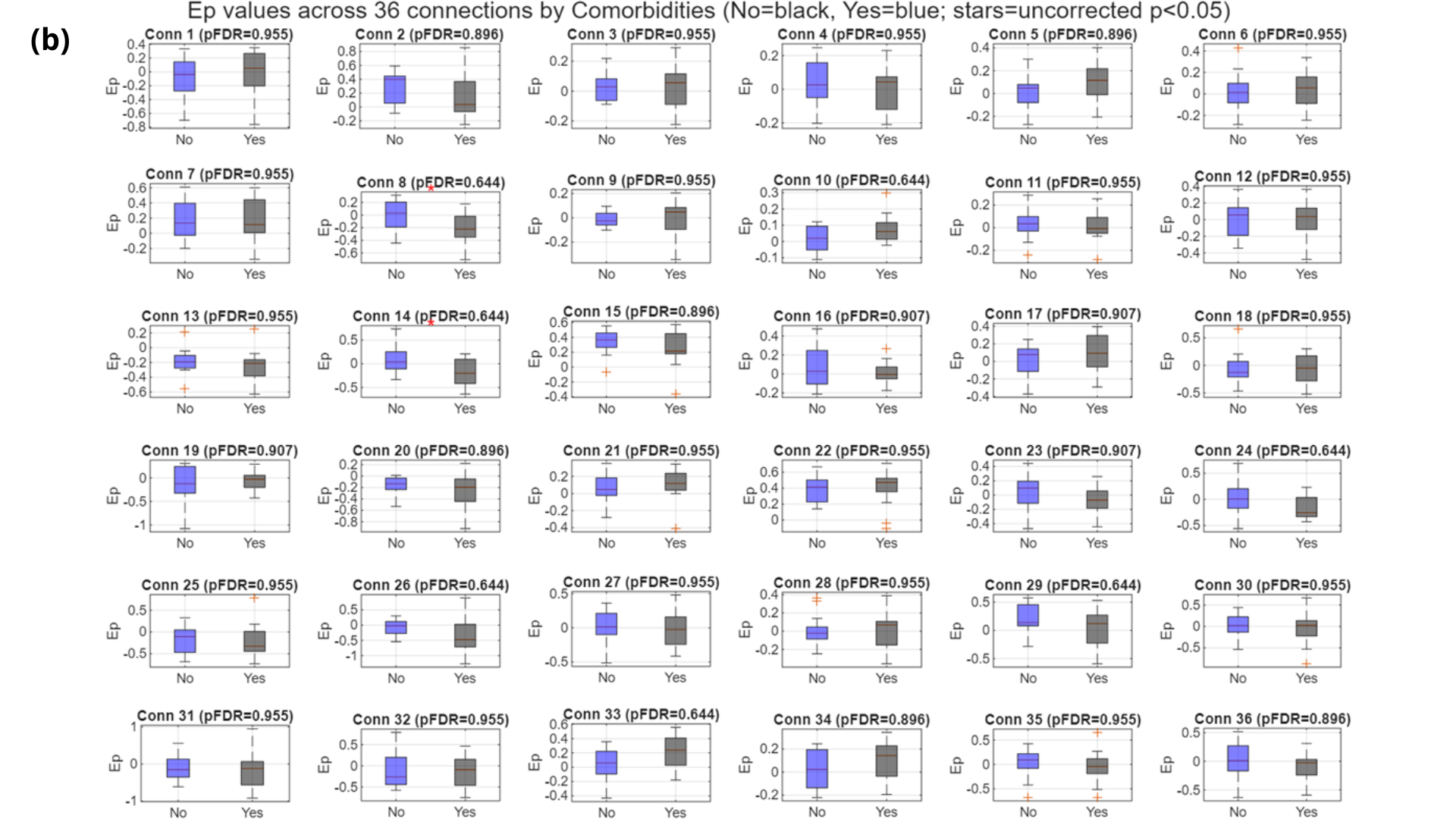


**Fig. 3b CAN connectivity changes in association to psychiatric comorbidities**. Distribution of Ep values across all 36 CAN connections for patients (n = 13) with and without psychiatric comorbidities (n = 13), in blue and black respectively. Group differences were tested using independent two-sample t-tests, with FDR correction applied across the 36 connections for comorbidity status. No effects survived FDR correction (pFDR ≥ 0.64). Boxplots illustrate the distribution of connectivity estimates across subgroups.


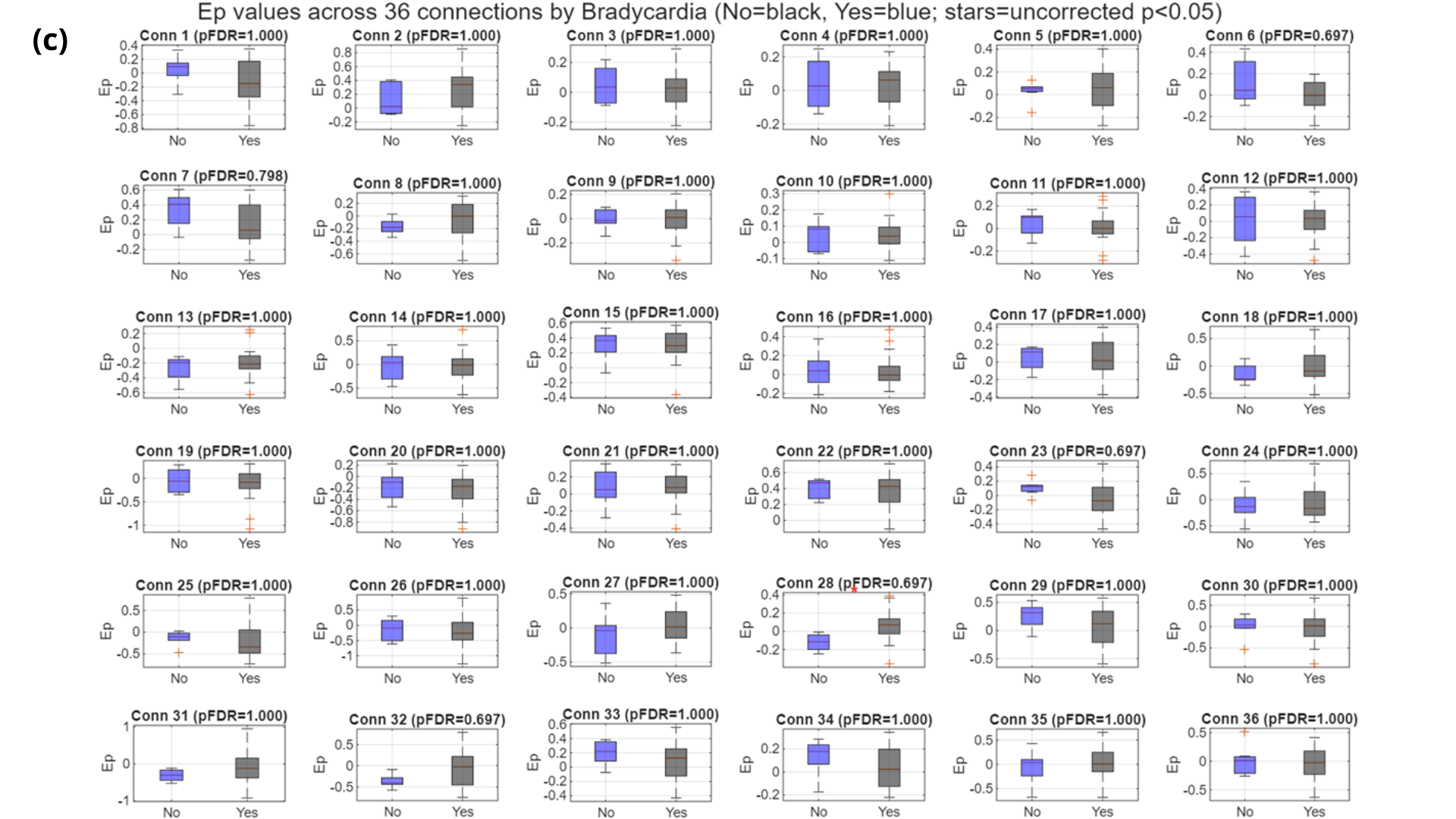


**Fig. S3c CAN connectivity changes in association to bradycardia.** Bradycardia was defined as resting heart rate < 60 bpm. Distribution of Ep values across all 36 CAN connections for patients with (n = 18) and without (n = 8) bradycardia, in blue and black respectively. Group differences were tested using independent two-sample t-tests, with FDR correction applied across the 36 connections for bradycardia. No effects survived FDR correction (pFDR ≥ 0.69). Boxplots illustrate the distribution of connectivity estimates across subgroups.


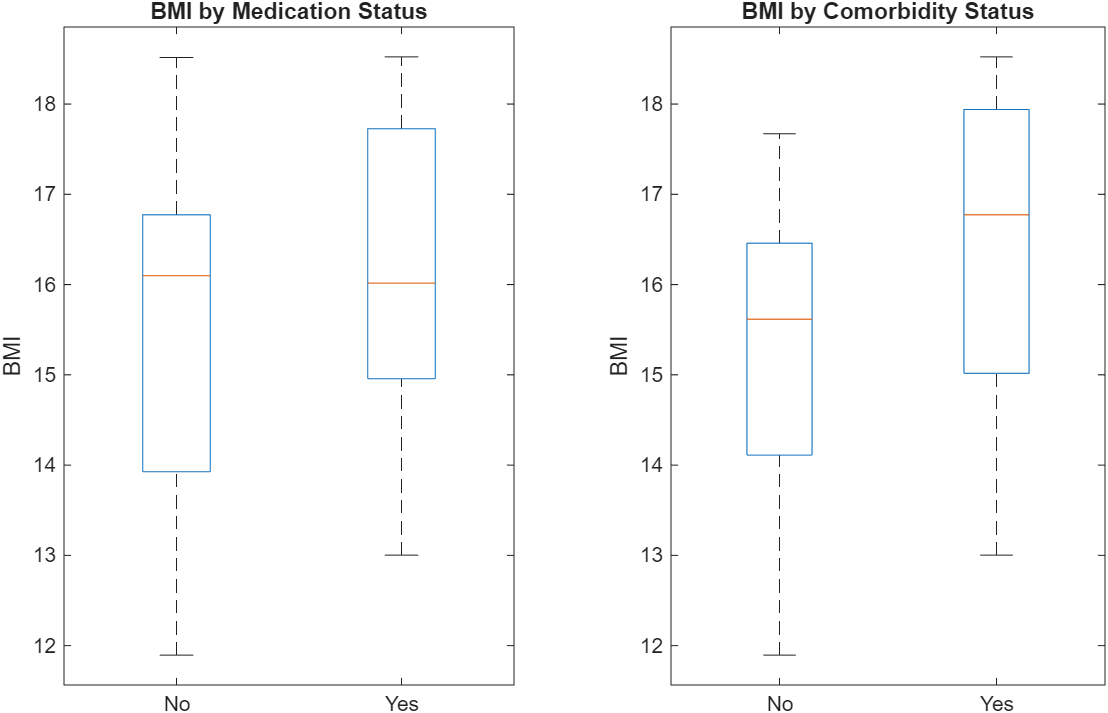


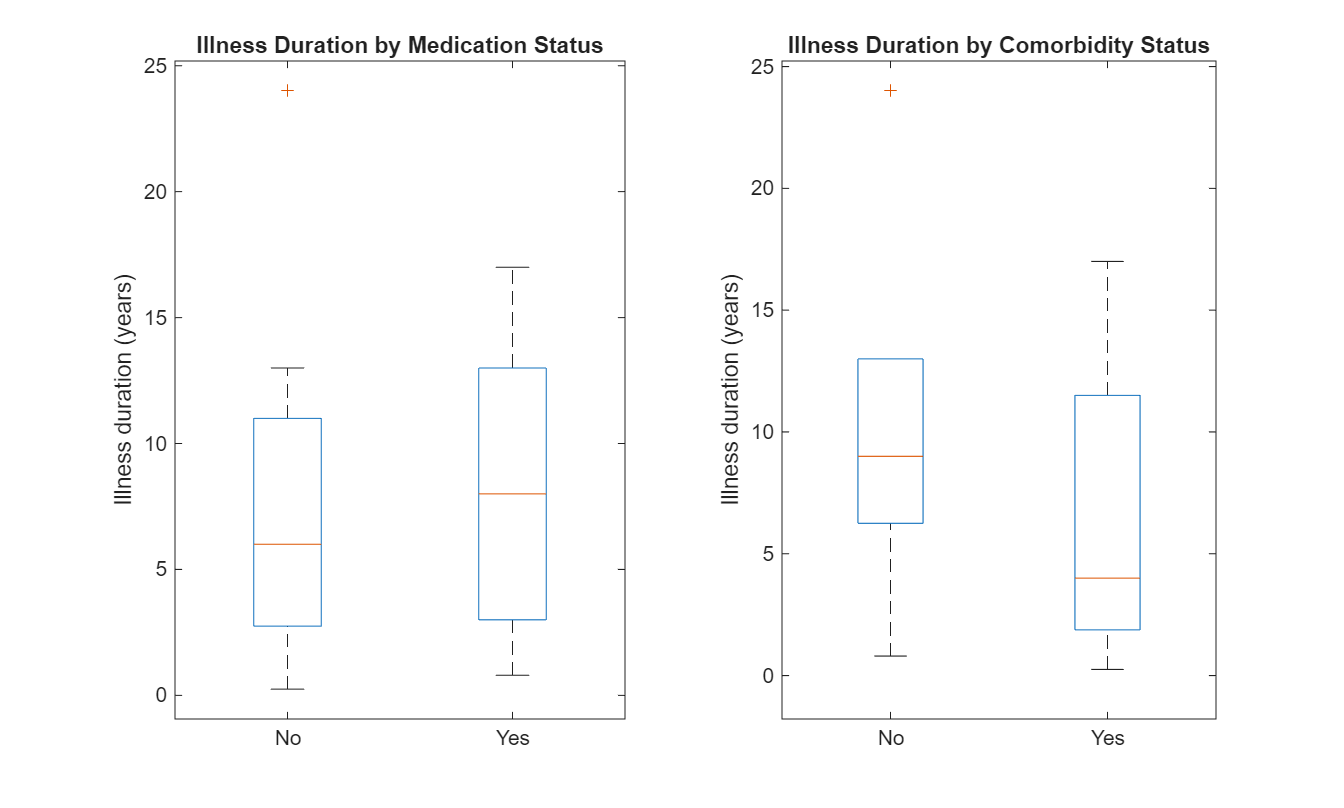


**Fig. S4 Comparison of body mass index (BMI) and illness duration between patient subgroups defined by medication status and psychiatric comorbidities**. Independent two-sample t-tests indicated no significant differences in BMI or illness duration between medicated and non-medicated patients (BMI: t = −0.86, p = 0.39; illness duration: t = −0.06, p = 0.94), nor between patients with and without comorbidities (BMI: t = −1.26, p = 0.21; illness duration: t = 1.15, p = 0.27). Illness duration analyses were conducted on the subset of patients with available duration estimates (n = 18). Boxplots show the distribution of BMI and illness duration across subgroups.

**Supplementary Analyses:**

**Age Effects on CAN connectivity**

As an exploratory analysis, we assessed whether effective connectivity within the central autonomic network (CAN) was associated with age. We computed Pearson correlations (FDR-corrected) between individual-level posterior estimates (Ep) from subject-specific DCMs and age. Correlation analyses focused on key connections previously identified as significant in the PEB within- and between-group analyses (PP > 0.99).

**Patients and Controls (HR associations):**
In AN patients, connectivity parameters linked to resting heart rate (self-connections of vmPFC, INS, AMY; HYPO → INS; BS → AMY; BS → HYPO; vmPFC → AMY; AMY → BS; HYPO → BS) showed no significant correlations with age (r = −0.05 to 0.16; uncorrected p = 0.10–0.87; pFDR > 0.71). Similarly, in healthy controls, connectivity parameters associated with HR (self-connections in INS/ACC, AMY → vmPFC, BS → vmPFC/ACC, AMY → INS, BS → HYPO, ACC → BS) were not significantly related to age (r = −0.07 to 0.23; uncorrected p = 0.08–0.62; pFDR > 0.45).

**Group × HR Interaction Effects:**
Connections showing significant PEB between-group HR interactions (self-connections of vmPFC/INS/AMY; vmPFC → INS; AMY → INS; BS → HYPO; ACC → BS; HYPO → BS) exhibited weak, non-significant associations with age in both patients (r = −0.007 to 0.12; uncorrected p = 0.13–0.87; pFDR > 0.90) and controls (r = −0.20 to 0.23; uncorrected p = 0.14–0.97; pFDR > 0.56).

**Group Differences (Diagnosis Effect):**
Exploratory correlations between age and CAN connections showing group differences (INS/HYPO self-connections, BS → vmPFC, vmPFC → ACC/HYPO, BS → ACC, vmPFC/ACC/INS → HYPO, INS → BS) were not significant in patients (r = −0.05 to 0.05; uncorrected p = 0.28–0.91; pFDR > 0.91) or controls (r = −0.02 to 0.37; uncorrected p = 0.01–0.87; pFDR > 0.09). One uncorrected correlation in controls (INS → HYPO, p = 0.01) did not survive FDR correction.

**Associations between resting heart rate, BMI, and symptom severity**

Finally, we examined whether resting heart rate (HR) was associated with body mass index (BMI) and symptom severity (EDI-2 total scores), separately in AN patients (n = 26) and healthy controls (HCs, n = 26). Resting HR was not significantly correlated with BMI in either group (AN: r = 0.36, p = 0.06; HCs: r = 0.05, p = 0.75). While a positive association between BMI and HR was observed at a trend level in the AN group, this effect did not reach statistical significance and should be interpreted cautiously. No association was observed in the control group. Similarly, resting HR was not significantly correlated with EDI-2 total scores in either AN patients (r = −0.21, p = 0.29) or HCs (r = 0.12, p = 0.45).
